# Multi-Path Interference Challenges and Suggested Solution for Correlation-Assisted Direct Time-of-Flight

**DOI:** 10.3390/s26092697

**Published:** 2026-04-27

**Authors:** Ayman Morsy, David Blinder, Maarten Kuijk

**Affiliations:** 1 Department of Electronics and Informatics, Vrije Universiteit Brussel, Pleinlaan 2, 1050 Brussels, Belgium; david.blinder@vub.be (D.B.); maarten.kuijk@vub.be (M.K.); 2 Interuniversity Microelectronics Centre (IMEC), Kapeldreef 75, B-3001 Leuven, Belgium

**Keywords:** multi-path interference, single-photon avalanche diode (SPAD), time of flight (ToF), light detection and ranging (LiDAR), depth sensing, resolving algorithm

## Abstract

The correlation-assisted direct time-of-flight (CA-dToF) pixel offers a promising approach for low-power consumption, ambient light robustness, and high-resolution depth sensing. However, as with other time-of-flight systems based on global phase estimation, its reliability is compromised by multi-path interference (MPI) of the emitted light, leading to depth estimation errors. In this work, the impact of MPI on CA-dToF pixel performance is systematically analyzed. Furthermore, a multi-frequency modulation algorithm is proposed to mitigate MPI effects. The proposed method improves depth accuracy and enhances the overall reliability of the CA-dToF system.

## 1. Introduction

Time-of-flight (ToF) technologies have been widely adopted across a range of applications, including autonomous driving systems in the automotive sector, machine vision for robotics and smart manufacturing, and facial recognition in consumer electronics such as laptops and smartphones [1,2,3,4]. The reliability of ToF systems is therefore critical for ensuring safety and security. A major challenge in ToF technology is mitigating multi-path interference (MPI), particularly under high ambient illumination. MPI arises from multiple reflections, including the direct reflection, which corresponds to the shortest path between the camera and the target object, and indirect (global) reflections, which result from stray light reflected from surrounding surfaces back to the camera. As the temporal separation between the direct and global reflection peaks decreases, distinguishing between them becomes increasingly difficult, thereby degrading depth accuracy.

Direct time-of-flight (dToF) is a sensing technique that determines depth by measuring the arrival time of incident photons and constructing a temporal histogram to estimate the time of reflected signals [4,5]. Owing to its ability to capture multiple return paths, advanced signal processing algorithms have been developed to distinguish between multi-path echoes, enabling robustness against MPI [6,7]. However, dToF systems are generally associated with high power consumption, which limits their scalability [8,9,10].

In contrast, indirect time-of-flight (iToF) systems employ modulated illumination in conjunction with in-pixel demodulation to estimate depth from the phase shift of the received signal, offering lower power consumption [4,11]. Nevertheless, iToF systems are constrained by limited unambiguous range, reduced performance under strong ambient illumination, and susceptibility to MPI effects. Several mitigation techniques can be used for indirect time-of-flight (iToF) and CA-dToF [11,12,13], such as, multiple-frequency demodulation methods and spot illumination [14,15,16,17,18,19,20].

The multiple-frequency demodulation method is mainly used in iToF, where multiple light modulations with harmonic frequencies are applied to the scene. The reflected light can be considered a superposition of the direct and MPI global reflections [19,20]. The detected signals can be demodulated using spectral estimation techniques [21]. This method showed reliable results in low-ambient-light conditions and the ability to avoid phase wrapping [19]. The output is further improved by increasing the number of harmonic frequencies applied and using macro-pixel architecture [18]. However, the system can be computationally intensive to operate on high-resolution images in real time [17,19]. Depending on the spectral estimation technique, ambient light shot noise can affect its performance. Therefore, deep learning has been implemented to correct MPI effects [22]. This method can be modified for CA-dToF operation to mitigate the MPI effect.

Another possible technique is spot-based illumination to create a 3D point cloud, in which the light is concentrated in a spot pattern at specific locations. Increasing the amplitude of directly reflected light relative to MPI reflections can minimize MPI effects. However, this method does not eliminate the MPI effects.

In this paper, we investigate the impact of MPI on the performance of the CA-dToF pixel and propose a method for distinguishing multiple reflection phases. Section 2 discusses the effect of MPI on CA-dToF operation. In Section 3, two experimental studies are presented to evaluate the influence of MPI on system performance. Then, a proposed method to resolve the MPI effect is discussed in Section 4, while Section 5 and Section 6 present the corresponding simulation results and discuss the limitations of the approach, respectively.

## 2. CA-dToF Operation Under Multi-Path Reflection

**Figure 1:**
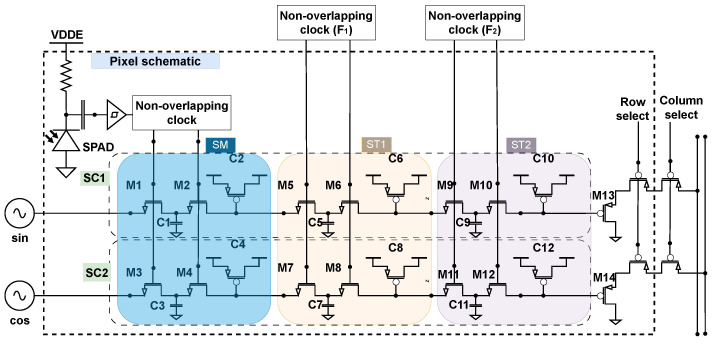
CA-dToF pixel schematic with multi-stage swiched capacitor [23].

The CA-dToF pixel is designed to average out ambient light by synchronizing orthogonal sinusoidal signals with a laser source, and sampling the detected light using a switched capacitor as presented in Figure 1 [23]. To simplify the pixel operation, the second and third stages ST1 and ST2 are closed by applying high DC voltage via F1 and F2, making the overall capacitance, $C_{total1}$ and $C_{total2}$, for the two analog channels, SC1 and SC2, equal to $C_{total1}=C_{2}+C_{5}+C_{6}+C_{9}+C_{10}$, $C_{total2}=C_{4}+C_{7}+C_{8}+C_{11}+C_{12}$, respectively. The sampled photon arrival time is detected by a SPAD sensor via the SM stage, and it is converted to a weighted voltage amplitude from a sinusoidal signal $V_{m,i}$, and exponentially averaged after multiple iterations *i* to $V_{i}$ via Equation (1), where $n_{av}=\frac{C_{1}+C_{total1}}{C_{1}}=\frac{C_{3}+C_{total2}}{C_{3}}$ is defined as the integration length [24]. The parameter $n_{av}$ determines the averaging power of the system, as for higher values of $n_{av}$, the system can reduce the detected voltage variance. The accumulated voltages are sampled via SC1 and SC2 analog channels and read out via two source followers (M13, M14), respectively.

(1) $$V_{i}=\frac{1}{n_{av}}\cdot V_{m,i}+\left(1-\frac{1}{n_{av}}\right)V_{i-1}$$

**Figure 2:**
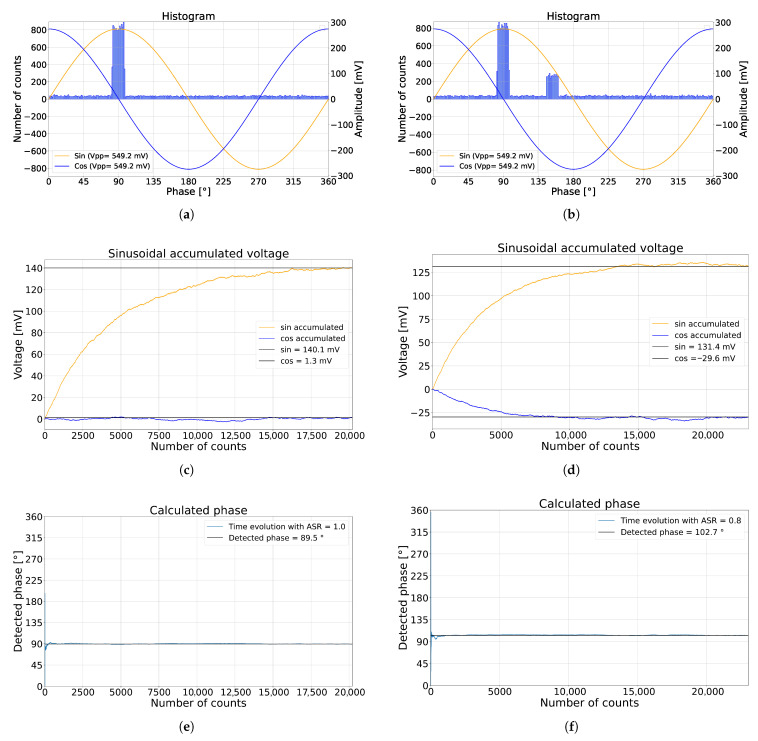
CA-dToF pixel simulation where (**a**,**b**) are the histograms from the detected events without and with multi-path reflection (MPR) with an amplitude ratio of 0.3, respectively. On the right side are the applied sinusoidal signals to the CA-dToF pixel. (**c**,**d**) are the voltage evolution of the analog channels SC1 and SC2. (**e**,**f**) are the calculated arrival times with an accuracy shift when MPI is introduced to the system.

The pixel performance is primarily determined by the amplitude of the reflected laser signal and the level of ambient illumination, such as sunlight or indoor lighting. The ratio of ambient to signal photons is called the ambient-to-signal ratio (ASR) and is a key parameter governing pixel performance [12]. This is simulated in the histogram shown in Figure 2a, where the laser-pulse arrival time is presented as a 90° phase shift across the full range of detection, with an ASR of 1. The use of phase shift comes from sampling the sinusoidal signal to determine the ToF. The applied sinusoidal voltage amplitude of 274.6 mV is sampled as presented in Figure 2c, where $n_{av}$ = 4000. The voltage evolution reaches equilibrium when it oscillates around a constant value. The voltage oscillation is primarily influenced by the ambient light shot noise. System confidence is defined as the magnitude of the summation of the detected sinusoidal signals. At equilibrium, it provides information on the detected ASR [12]. The detected phase is calculated and presented in Figure 2e. When MPI is absent, the detected phase corresponds to the ground truth of the detected object.

The effect of MPI is illustrated in Figure 2b, which presents a simulation featuring two peaks at 90° and 153°. The first peak corresponds to direct light transport from the laser and matches the ground truth with the earliest arrival time. In contrast, the second peak corresponds to global light transport caused by indirect reflections. The second MPI peak count is 0.3 from the direct peak, with an ASR of 0.8. This study focuses on one direct light peak and one global light peak. CA-dToF pixel performance relies on the number of counts of the sampled sinusoidal signal. Therefore, the MPI/laser ratio, defined in Equation (2), indicates the MPI’s effect on system performance.

(2) $$\mathrm{MPI/laser}=\frac{\mathrm{MPI}\;\mathrm{photons}}{\mathrm{Laser}\;\mathrm{photons}}$$

Figure 2d shows the accumulated voltage between the two peaks, indicating a phase error, illustrated in Figure 2f. The results demonstrate that the detected MPI peak introduces accuracy errors, thereby degrading the reliability of the CA-dToF pixel.

To investigate the parameters affecting the MPI in CA-dToF pixel performance, the system is simulated over multiple ASR levels, with different MPI/laser ratios, with fixed laser and MPI arrival times of 90° and 153°, respectively. Each data point is the average of 200 simulations, using an integration length $n_{av}$ of 4000 and 5 photons per cycle over 10,000 cycles. Under the used simulation condition, SPAD deadtime effect is negligible. However, SPAD deadtime effects are further analyzed in [23].

**Figure 3:**
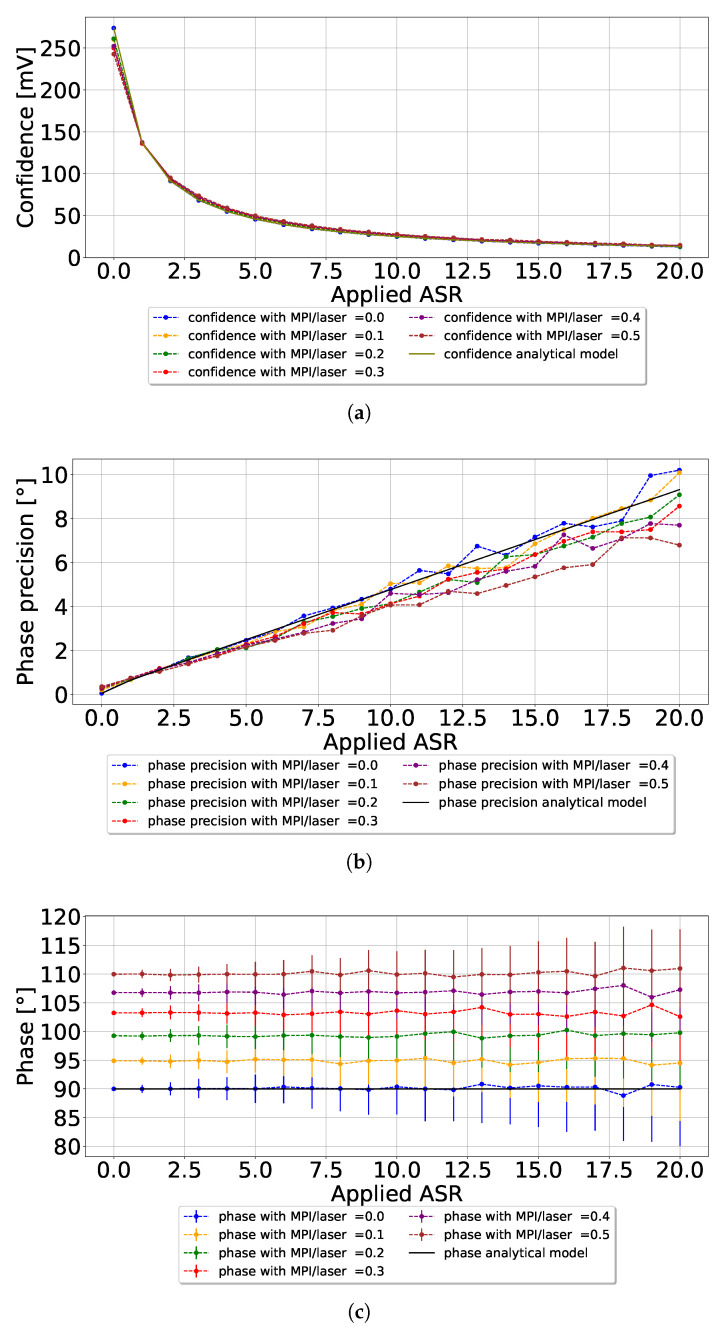
MPI effect on CA-dToF pixel performance; (**a**) is the confidence output for different ASR and MPI/laser ratios; (**b**) is the associated phase precision; (**c**) is the phase degradation due to MPI, with increased phase precision due to ASR.

The simulation results are compiled in Figure 3. In previous publications, the pixel operation was analytically modeled and verified with simulation and experimental work [12,25]. Therefore, the CA-dToF analytical model is used to understand the MPI effect. Figure 3a shows that the measured confidence levels closely align with the analytical model across different ASRs, with deviations of less than 10%. As illustrated in Figure 3b, similar behavior is also observed for phase precision. However, the phase accuracy shows significant and consistent deviation across different MPI/laser ratios, as illustrated in Figure 3c.

The results underscore the critical influence of MPI on CA-dToF pixel performance. While phase precision and confidence remain primarily consistent with theoretical predictions, phase accuracy is significantly impacted by variations in MPI/laser ratios. Furthermore, the phase error is independent of the ASR applied to the system. This highlights the need for strategies to mitigate MPI-induced errors in ToF systems to ensure reliable and accurate depth sensing across different ASR levels.

## 3. MPI Test Cases for CA-dToF

Several experimental configurations can be used to evaluate MPI, including diffusive bulk scattering, multi-surface reflections, and inter-reflections within a scene [14,26,27]. To demonstrate MPI in the context of CA-dToF, experiments involving scene inter-reflections and multi-surface reflections were conducted.

**Figure 4:**
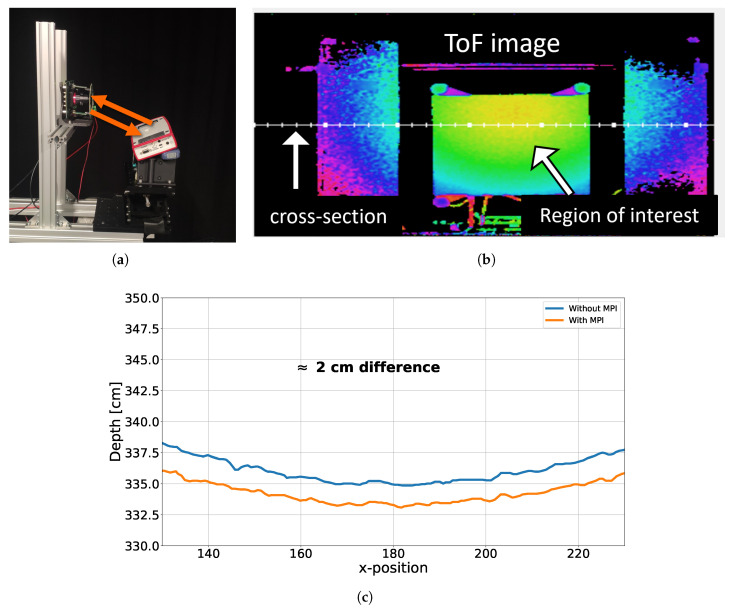
Inter-reflection in a scene on CA-dToF; (**a**) is a QVGA camera of CA-dToF with a reflective object underneath; (**b**) the detected object; (**c**) the detected object distance with and without MPI.

In the inter-reflection experiment, an additional object was positioned farther from the target, so that its reflected light influenced the direct reflection from the target. The experimental setup is illustrated in Figure 4a, where a CA-dToF QVGA camera is used [25]. In this pixel operation, stages ST1 and ST2 in Figure 1 are used with modulation frequencies of F1 = 1 MHz and F2 = 10 kHz to reduce the systematic noise as published previously in [23]. In the absence of inter-reflections, the target object, shown in Figure 4b, was measured at a distance of 335 cm, as presented in Figure 4c. When the additional object was introduced, the perceived distance of the target decreased to around 333 cm, corresponding to a 2 cm shift from its original position, as shown in Figure 4c. The curved detected distance is due to Petzval field curvature, a lens aberration.

**Figure 5:**
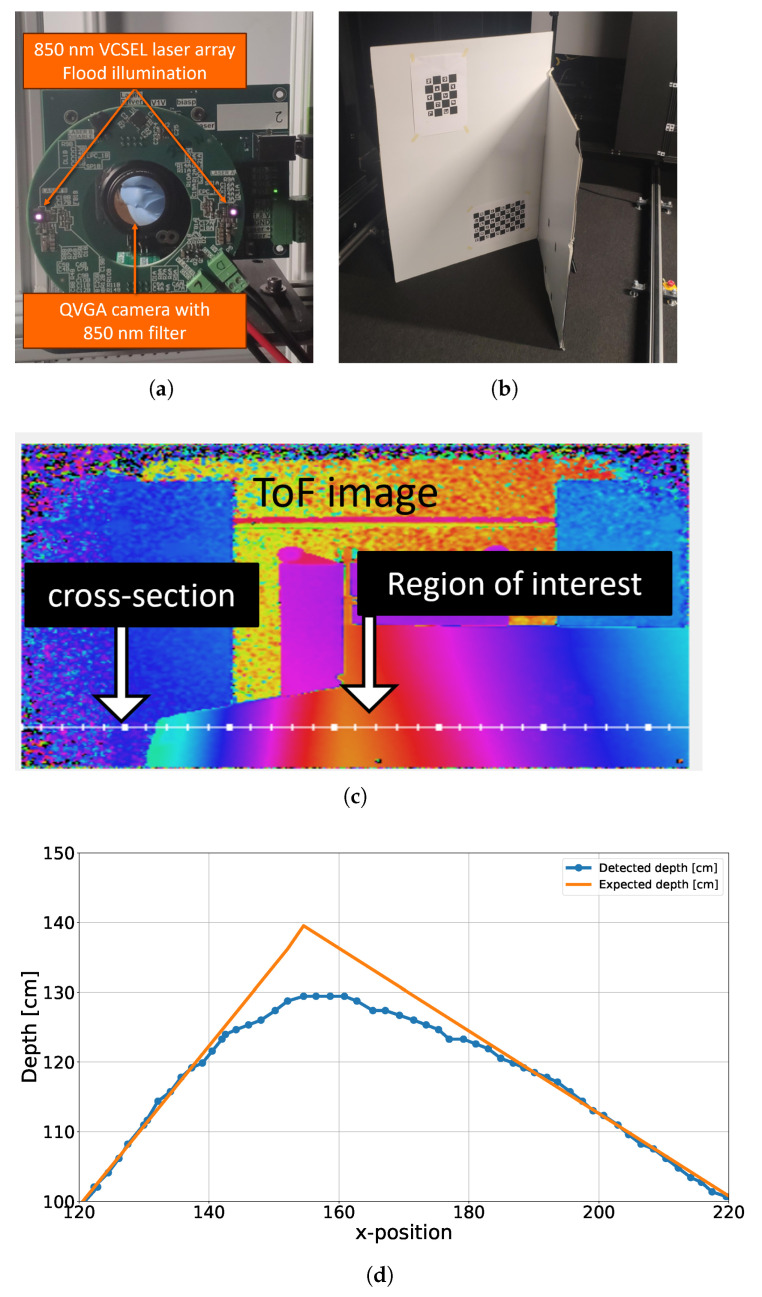
MPI experimental effect on CA-dToF pixel performance; (**a**) is a QVGA camera of CA-dToF, where flood illumination is used; (**b**) is a two-reflective object creating a corner; (**c**) is the ToF image of the scene with a cross section of the ToF; (**d**) is the depth profile of the corner with a predicted depth (in orange) and measured depth (in blue), where the corner information is lost.

In the multi-surface reflection experiment, two planar surfaces were arranged to form a sharp, corner-like geometry and were positioned in front of the CA-dToF QVGA camera, as shown in Figure 5a [25]. Multiple reflections between adjacent surfaces before reaching the camera result in a shorter apparent path length than the true geometric distance, effectively blurring the perceived corner. The experimental corner configuration is illustrated in Figure 5b. By capturing the scene and analyzing the corner profile using a cross-sectional line, as depicted in Figure 5c, a noticeable degradation in corner sharpness is observed, as shown in Figure 5d.

## 4. Proposed Solver Method

To tackle the MPI in CA-dToF, the spectral estimation technique [19] is adapted to CA-dToF. It uses an analytical model to predict the amplitudes and phases of detected peaks. Assuming a Gaussian pulse with standard deviation σ, peak amplitude *a* and time delay *x*, its integration with a (co)sine wave yields:

(3) $$\int_{-\infty }^{\infty }a\cdot e^{-\frac{{\left(u-x\right)}^{2}}{2\;\sigma^{2}}}\cos\left(u\right)\;du=a\sqrt{2\pi }\sigma \;e^{-\frac{\sigma^{2}}{2}}\cos\left(x\right)=a^{\prime }\cos\left(x\right).$$

Namely, the peak width proportional to σ only scales all measurements by a constant, leaving the overall algorithm unmodified. We thus proceed by treating the peaks as ideal delta functions in the remainder of this section. The model assumes two peaks detected in phases *x* and *y* and amplitudes *a* and *b*, respectively. However, the model can be generalized to higher-order peaks.

### 4.1. Problem Statement

Consider a system with two unknown peaks at positions *x* and *y* and unknown amplitudes *a* and *b*, respectively. For every pixel, we make a small set of measurements $c_{j},j\in \left\{1,2,\ldots ,2N\right\},N\in N$ such that

(4) $$\begin{array}{c} \left\{\begin{array}{c} c_{1}=a\cos\left(x\right)+b\cos\left(y\right)\\ c_{2}=a\sin\left(x\right)+b\sin\left(y\right)\\ c_{3}=a\cos\left(2x\right)+b\cos\left(2y\right)\\ c_{4}=a\sin\left(2x\right)+b\sin\left(2y\right)\\ \ldots \\ c_{2N-1}=a\cos\left(Nx\right)+b\cos\left(Ny\right)\\ c_{2N}=a\sin\left(Nx\right)+b\sin\left(Ny\right) \end{array}\right. \end{array}$$

Note that the system is highly nonlinear and ill-posed. For example, it is symmetrical in the peak coordinates, i.e., swapping *a* and *x* with *b* and *y* has no effect. Moreover, whenever x≈y or a≈0 or b≈0, ambiguities may arise due to noise and measurement errors.

We simplify the equations in several steps. First, we can rewrite them in complex form using Euler’s formula exp(iz)=cos(z)+isin(z):

(5) $$\begin{array}{c} \left\{\begin{array}{c} p_{1}=c_{1}+ic_{2}=a\exp\left(ix\right)+b\exp\left(iy\right)\\ p_{2}=c_{3}+ic_{4}=a\exp\left(2ix\right)+b\exp\left(2iy\right)\\ \ldots \\ p_{N}=c_{2N-1}+ic_{2N}=a\exp\left(Nix\right)+b\exp\left(Niy\right) \end{array}\right. \end{array}$$

If we treat the right-hand sides as complex-valued unknowns α=aexp(ix) and β=bexp(iy), we get

(6) $$\begin{array}{c} \left\{\begin{array}{c} p_{1}=\alpha +\beta \\ p_{2}=\frac{\alpha^{2}}{\left|\alpha \right|}+\frac{\beta^{2}}{\left|\beta \right|}\\ \ldots \\ p_{N}=\frac{\alpha^{N}}{{\left|\alpha \right|}^{N-1}}+\frac{\beta^{N}}{{\left|\beta \right|}^{N-1}} \end{array}\right. \end{array}$$

where |·| is the absolute value or magnitude of the complex unknowns α,β∈C. We further simplify the system by a change of variables $z=\frac{\alpha }{p_{1}}$ and $w=\frac{\beta }{p_{1}}$, i.e., scaling by $p_{1}$, giving

(7) $$\begin{array}{c} \left\{\begin{array}{c} 1=z+w\\ p_{2}=\frac{z^{2}p_{1}^{2}}{\left|zp_{1}\right|}+\frac{w^{2}p_{1}^{2}}{\left|wp_{1}\right|}\\ \ldots \\ p_{N}=\frac{z^{N}p_{1}^{n}}{{\left|zp_{1}\right|}^{N-1}}+\frac{w^{N}p_{1}^{N}}{{\left|wp_{1}\right|}^{N-1}} \end{array}\right. \end{array}$$

From the first equation 1=z+w, we can substitute w=1−z into this system, reducing the problem to solving for a single variable z∈C. We also bring the $p_{1}$ constants to the left-hand sides and absorb them into new constants $q_{j},j\in \left\{2,\ldots ,N\right\}$, resulting in

(8) $$\left\{\begin{array}{c} q_{2}=p_{2}\frac{\left|p_{1}\right|}{p_{1}^{2}}=\frac{z^{2}}{\left|z\right|}+\frac{{\left(1-z\right)}^{2}}{\left|1-z\right|}\\ \ldots \\ q_{N}=p_{N}\frac{{\left|p_{1}\right|}^{N-1}}{p_{1}^{N}}=\frac{z^{N}}{{\left|z\right|}^{N-1}}+\frac{{\left(1-z\right)}^{N}}{{\left|1-z\right|}^{N-1}} \end{array}\right.$$

In summary, for 2N measurements, we have to solve a complex-valued system of N−1 equations in *z*. By multiplying the solution for *z* by $p_{1}$, we can retrieve α and β, after which we can find the unknowns {a,b,x,y} by extracting the magnitude and complex arguments.

### 4.2. Solving the System of Equations

We want to find the “best” solution for *z* in the equation system

(9) $$q_{j}=\frac{z^{j}}{{\left|z\right|}^{j-1}}+\frac{{\left(1-z\right)}^{j}}{{\left|1-z\right|}^{j-1}},\;j\in \left\{2,\ldots ,N\right\}$$

where the $q_{j}$ are obtained from our measurements, with noise and potential measurement errors. This amounts to solving a nonlinear least-squares problem, which we do using the Levenberg–Marquardt algorithm. It can be viewed as an interpolation between the Gauss-Newton algorithm and the gradient descent method, making it more robust. In our experiments, we run the algorithm 10 times with different random starting values and keep the best solution, increasing the probability of finding the global minimum.

## 5. Simulation Results

The statistical and analytical models of CA-dToF pixels have been experimentally validated in previous studies [12,25]. Accordingly, the statistical model can be reliably employed to investigate the impact of MPI on CA-dToF systems and to evaluate the performance of the proposed peak-resolution method.

**Figure 6:**
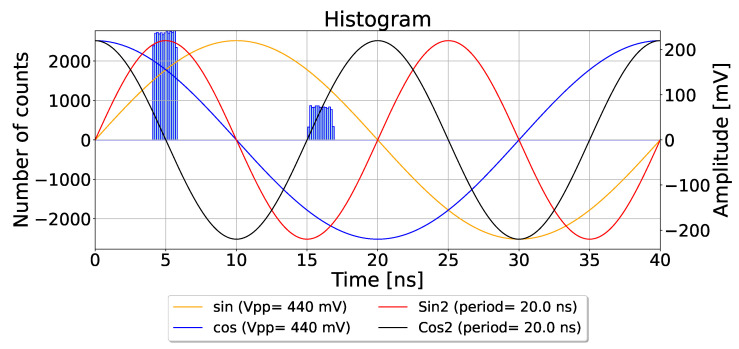
The histogram from the detected events with MPI/laser ratio of 0.3, applying two harmonic sinusoidal periods.

**Figure 7:**
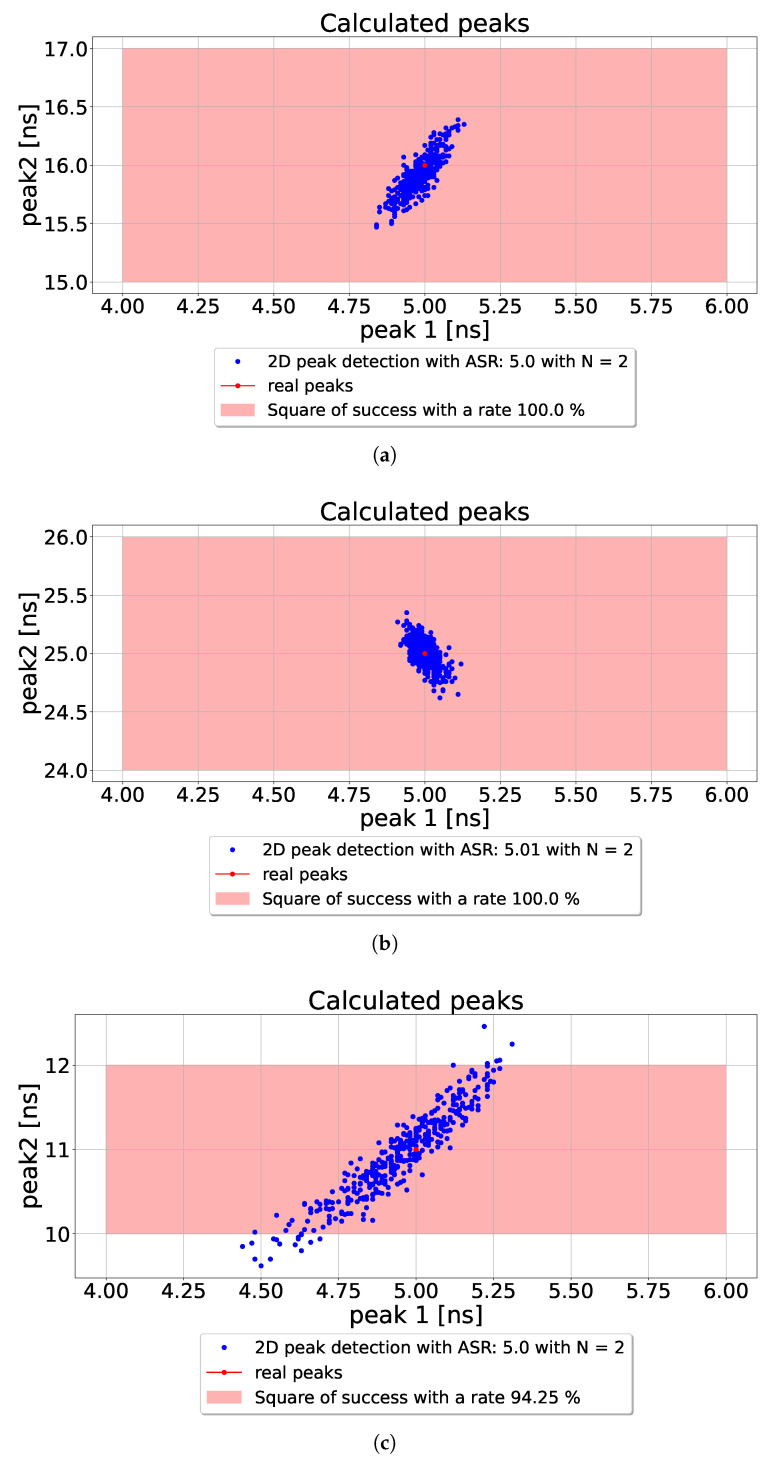
The MPI resolver algorithm results repeated 400 times with ASR of 5 and MPI/laser ratio of 0.3 (N indicates the number of harmonics. In this case, it is two); (**a**) is the distribution of the detected phase when the second peak is detected at 16 ns; (**b**) is the result when the second peak is detected at 25 ns, which reduces the impact of phase wrapping. (**c**) is the result when the temporal separation between the two peaks is set to three times the laser pulse width, ensuring minimal overlap and improving peak resolution.

The simulation conditions for (MPI) are illustrated in Figure 6, where two distinct peaks are detected at 10 ns and 16 ns using the CA-dToF pixel with a laser pulse width of 1.7 ns. Two harmonic sinusoidal modulation periods of 40 ns and 20 ns are applied. The amplitude response is sampled 400 times, and for each sample, the resulting amplitudes are used as input to the algorithm proposed in Section 4.2. The results are shown in Figure 7a, where the red dot denotes the actual locations of the peaks, and the blue dots represent the calculated values. A red square centered at the actual peaks, with a width of ±1 ns, defines the success region. The algorithm demonstrates consistent performance, with the standard deviation of the estimates influenced by ambient light conditions and the integration length.

Notably, the algorithm can detect a second peak even beyond the shortest modulation period of 20 ns, with a comparable level of precision, as shown in Figure 7b. This indicates that the proposed method effectively reduces phase wrapping errors. Applying higher-order harmonics to the system is possible, but it reduces the frame rate and increases motion blur. Furthermore, we did not find a significant gain from using higher-order harmonics in the system. Therefore, we decided to analyze a two-harmonic system for two peaks.

The ASR limits the system’s ability to resolve two closely spaced peaks. Higher ASR values impair the algorithm’s capacity to distinguish between closely spaced peaks. Empirically, the minimum resolvable peak separation should be approximately three times the laser pulse width. A separation below this threshold, especially under high ASR conditions, increases the risk of peak interference. This behavior is demonstrated in Figure 7c, where the algorithm is tested with peak separations at 5 ns and 11 ns.

Table 1 summarizes the standard deviation across different conditions, showing a comparable deviation for each peak. The source of this deviation lies in the system’s inherent nonlinearity: even minor noise in the detected amplitudes from ASR can significantly affect the algorithm’s output. This effect is particularly evident in Figure 7c, where the close proximity of the two peaks leads to increased nonlinearity and a corresponding rise in standard deviation.

**Figure 8:**
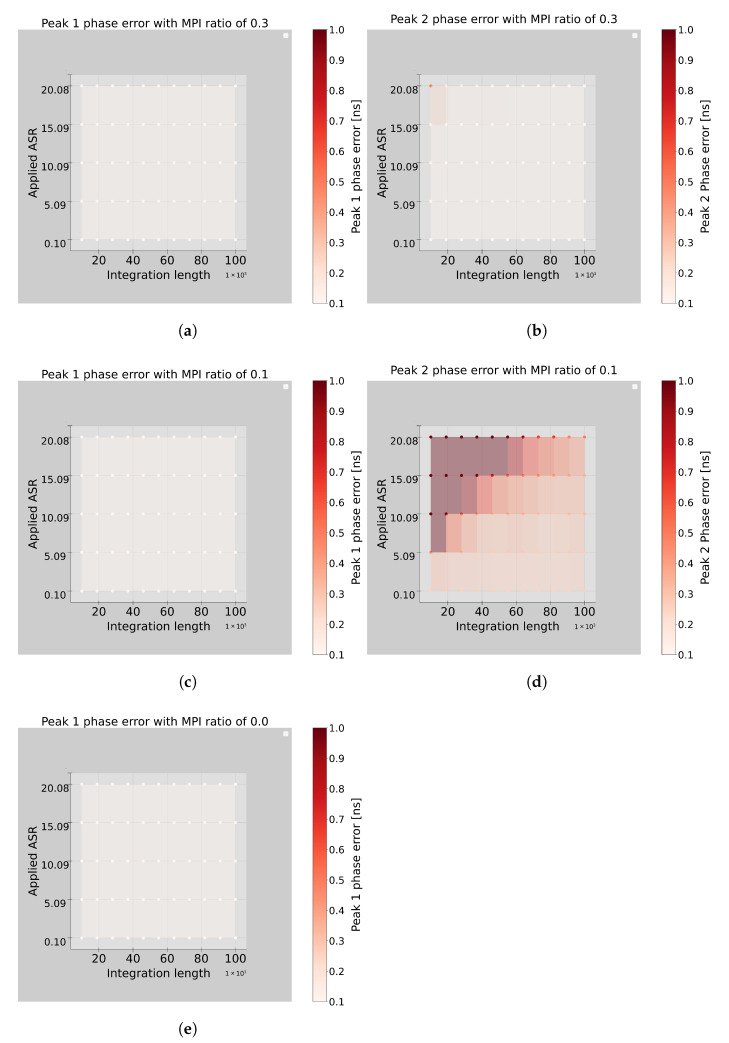
The detected phase error of the two peaks generated from the MPI resolver algorithm for different integration lengths and ASRs; (**a**,**b**) are the first and second peak phase error with MPI/laser ratio of 0.3, respectively. (**c**,**d**) are the first and second peak phase errors with an MPI/laser ratio of 0.1. (**e**) is the first peak phase error when no MPI exists in the system.

To comprehensively assess the algorithm’s behavior in different environmental conditions, simulations are conducted by systematically scanning a range of ASR values and integration lengths. For performance characterization, the difference between the actual peak position and the mean of the detected peak is used as the primary metric. Each configuration is simulated 400 times to compute the mean and standard deviation. Results for two multi-path interference (MPI) to laser amplitude ratios—0.3 and 0.1—are shown in Figure 8.

The detection of the first peak demonstrates reliable phase accuracy, with phase errors as low as 0.1 ns, regardless of MPI ratio, ASR levels, or integration lengths, as shown in Figure 8a,c. In contrast, the second peak exhibits greater sensitivity to these parameters. Higher MPI ratios lead to more reliable second peak detection, while low integration lengths combined with high ASR values significantly degrade performance, as illustrated in Figure 8b,d.

**Figure 9:**
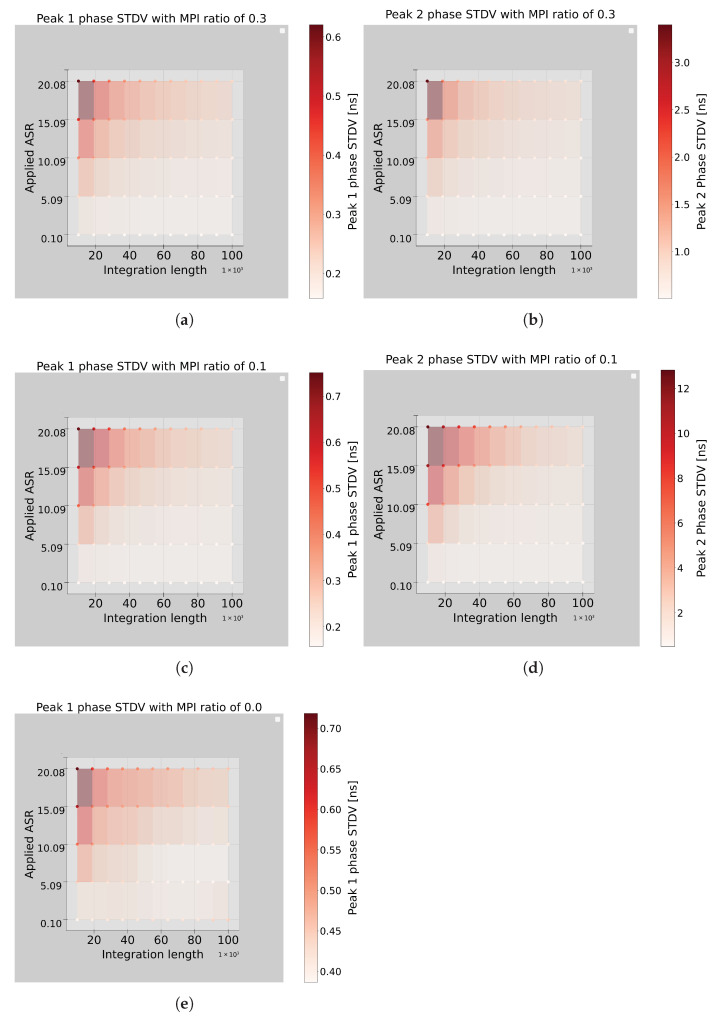
The detected phase standard deviation of the two peaks generated from the MPI resolver algorithm for different integration lengths and ASRs; (**a**,**b**) are the first and second peak phase standard deviations with MPI/laser ratio of 0.3, respectively. (**c**,**d**) are the first and second peak phase standard deviations with MPI/laser ratio of 0.1. (**e**) is the first peak phase standard deviation when no MPI exists in the system.

The standard deviation of the detected phases follows a consistent trend, as shown in Figure 9. For the first peak, phase precision ranges from 0.1 ns to 0.7 ns, depending primarily on ASR and integration length (Figure 9a,c). The second peak, however, exhibits greater variability, strongly influenced by the MPI ratio, ASR, and integration length. At an MPI ratio of 0.3, the standard deviation ranged from 0.5 ns to 3.4 ns under high ASR and short integration length (Figure 9b). With a lower MPI ratio of 0.1, performance worsens considerably, with standard deviations spanning from 0.5 ns to 12.8 ns (Figure 9d), indicating the algorithm’s limitations in resolving low-amplitude secondary peaks under challenging conditions.

To assess the algorithm’s ability to detect MPI, simulations are also performed under conditions without MPI, presented in Figure 8e. The results show that the first peak could still be accurately detected with sub-nanosecond phase resolution, reinforcing the method’s reliability in identifying the presence or absence of MPI. The second peak phase was not presented because it yielded ambiguous results. The standard deviation of the first peak detection in the absence of MPI ranges from 0.4 ns to 0.7 ns, as shown in Figure 9e. For enhanced MPI detection, longer integration lengths are recommended. On average, the algorithm required 20 iterations to extract the two peaks.

In summary, the algorithm reliably detects the first peak, achieving high accuracy and moderate phase noise. However, detection of the second peak is highly sensitive to the MPI ratio, ASR, and integration length. To ensure consistent dual-peak detection, increasing the integration length is recommended, particularly under high ASR scenarios.

## 6. Limitations and Future Improvements of the Proposed Algorithm

The proposed algorithm assumes an ideal laser pulse modeled as a Gaussian function, thereby making its performance independent on the actual pulse shape. While this assumption simplifies the analytical framework, it does not fully reflect real-world conditions, where laser pulses exhibit finite temporal widths. Following Equation (3), the temporal distribution of the emitted laser pulse can affect the algorithm’s reliability, with broader pulses introducing additional uncertainty and increasing depth estimation errors. Consequently, the achievable resolution is fundamentally constrained by the laser pulse width. Simulation observations indicate that the algorithm can reliably distinguish between two peaks separated by at least three times the pulse width.

Although the current implementation focuses on the analysis of two peaks, the framework can, in principle, be extended to accommodate higher-order reflections by incorporating additional frequency components. Nevertheless, our investigation into two-peak resolution suggests that including higher-order frequency terms does not yield a significant improvement in phase estimation accuracy. Instead, it leads to increased computational complexity and reduced frame rates, as higher modulation frequencies are required. The overall processing time per frame depends on the hardware architecture and level of parallelization; however, the per-pixel iterative nature of the algorithm indicates that computational cost scales linearly with the number of pixels. These trade-offs highlight the need for careful optimization when extending the algorithm to more complex multi-path scenarios.

The algorithm assumes that the higher-order frequency components exhibit identical amplitudes and phase shifts. However, in practical implementations, particularly when on-chip triangular signals are generated, both amplitude and phase may vary with frequency due to the RC effect of the transmission lines. While no fundamental limitations are anticipated, challenges may arise in large pixel arrays, where phase variations across the array can occur due to signal propagation delays. Consequently, careful design of the signal generation circuitry and signal distribution is required to ensure consistent amplitude and phase characteristics across the employed frequency components, depending on the desired detection range.

The CA-dToF camera has demonstrated reliable performance under both simulated indoor and outdoor conditions [24,25]. While outdoor operation is primarily constrained by the need to suppress strong ambient sunlight, indoor environments pose a greater MPI challenge. In such settings, multiple surfaces can contribute reflected signals to the same pixel, thereby degrading performance, as experimentally demonstrated in Section 3. Due to the relatively low ambient light levels in indoor environments, we expect the proposed algorithm to operate reliably. The simulation analysis in this work focuses on a single MPI component, as higher-order reflections are expected to have negligible amplitudes compared to the direct and first-order global reflections.

Overall, while the proposed algorithm demonstrates effective performance under the considered conditions, its practical applicability is constrained by the finite laser pulse width, limitations in amplitude measurement, and computational constraints, warranting further investigation and refinement. The proposed method demonstrates the feasibility of adapting existing MPI mitigation approaches developed for iToF systems to CA-dToF operation.

## 7. Conclusions

This study investigated the impact of multipath interference (MPI) on CA-dToF performance through both simulation and experimental validation. The results demonstrate that MPI degrades depth detection accuracy, largely independent of ambient illumination conditions, without a significant observable change in distance precision.

To address this challenge, a peak-resolving algorithm was proposed and evaluated through simulation, demonstrating its effectiveness in mitigating MPI-induced errors. The algorithm’s limitations were also analyzed, including its dependence on pulse characteristics and its potential for extension to higher-order MPI scenarios. The result suggests that approaches developed for iToF systems can be extended to CA-dToF, and simulation results confirm the feasibility of this adaptation when appropriately tailored to the CA-dToF operation.

Overall, this work contributes to improving the robustness and reliability of CA-dToF systems and represents a step toward more accurate and dependable time-of-flight depth sensing. 

## Figures and Tables

**Table 1:** Parameters used for generating each figure in Figure 7 with the corresponding results. The ASR for all figures is 5 with an integration length of 10^5^ and a pulse width of 1.7 ns.

Figure	Peak 1 [ns]	Peak 2 [ns]	Peak 1 STDV [ns]	Peak 2 STDV [ns]
Figure 7a	5	16	0.05	0.16
Figure 7b	5	25	0.03	0.12
Figure 7c	5	11	0.17	0.52

## Data Availability

The original contributions presented in this study are included in the article. Further inquiries can be directed to the corresponding authors.

## Abbreviations

The following abbreviations are used in this manuscript:

CA-dToF	Correlation-Assisted Direct Time-of-Flight
iToF	Indirect Time-of-Flight
MPI	Multi-Path Interference
EWMA	Exponential Weighted Moving Average
ASR	Ambient-to-Signal Ratio
